# Supplementary material for: Chromothripsis-like patterns are recurring but heterogeneously distributed features in a survey of 22,347 cancer genome screens
Source: BMC Genomics. 2014 Jan 29;15:82. doi: 10.1186/1471-2164-15-82 (PMC3909908; doi:10.1186/1471-2164-15-82)
Supplement: Additional file 1 — Supplementary figures and tables. Figure S1. Scatter plot of the training set. Figure S2. The positive training set and CTLP detection algorithm performances. Figure S3: Scatter plot of CTLP candidates. Figure S4. Kaplan-Meier survival curves for CTLP versus non-CTLP cases in specific cancer types. Figure S5. An example of the platform resolution based simulation using data from an Affymetrix SNP6 array (~1.8 million probes). Figure S6. CTLP detection sensitivity of simulated platform resolutions. Table S1. Overview of input dataset. Table S7. Demographic and clinico-pathologic characteristics of input and CTLP samples. Table S8. Sizes of sliding windows for the scan-statistic based algorithm. [file 1471-2164-15-82-S1.pdf]

Supplementary Information

Chromothripsis-like patterns are recurring but heterogeneously distributed features in a survey of 22,347 cancer genomes

Haoyang Cai[1,2], Nitin Kumar[1,2], Homayoun C. Bagheri[3], Christian von Mering[1,2], Mark D. Robinson[1,2], and Michael Baudis[1,2]

[1]Institute of Molecular Life Sciences, University of Zurich, Zurich, Switzerland
[2]Swiss Institute of Bioinformatics, University of Zurich, Zurich, Switzerland
[3]Institute of Evolutionary Biology and Environmental Studies, University of Zurich, Zurich, Switzerland

1. Supplementary Figures

Supplementary Figure 1 **page 3**

Scatter plot of the training set.

Supplementary Figure 2 **page 11**

The positive training set and CTLP detection algorithm performances.

Supplementary Figure 3 **page 12**

Scatter plot of CTLP candidates.

Supplementary Figure 4 **page 13**

Kaplan-Meier survival curves for CTLP versus non-CTLP cases in specific cancer types.

Supplementary Figure 5 **page 14**

An example of the platform resolution based simulation from Affymetrix SNP6 array (1.8M).

Supplementary Figure 6 **page 15**

CTLP detection sensitivity of simulated platform resolutions.

2. Supplementary Tables

Supplementary Table 1 **page 16**

Overview of input dataset

Supplementary Table 7 **page 17**

Demographic and clinicopathologic characteristics of input and CTLP samples

Supplementary Table 8 **page 18**

Sizes of sliding windows for the scan-statistic based algorithm

Additional File 1: Supplementary Figures and Tables

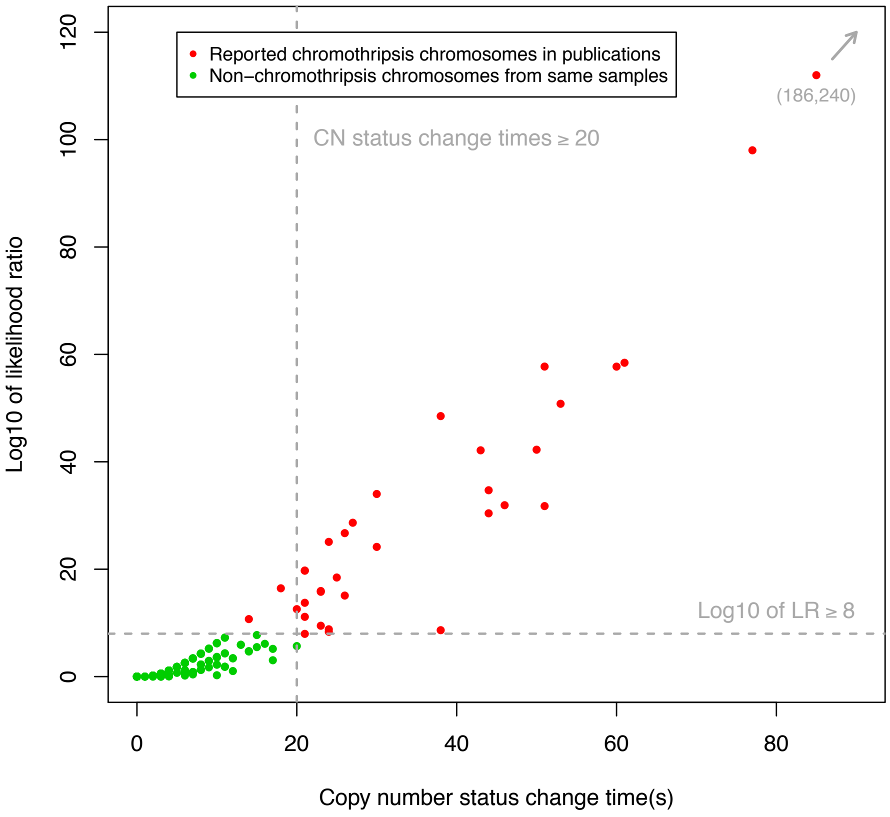

Figure S1. Scatter plot of the training set. Copy number status change times compared to the likelihood ratio. Each point represents the window with the highest LR for each chromosome. The dashed lines indicate the selected thresholds. CN, copy number; LR, likelihood ratio.

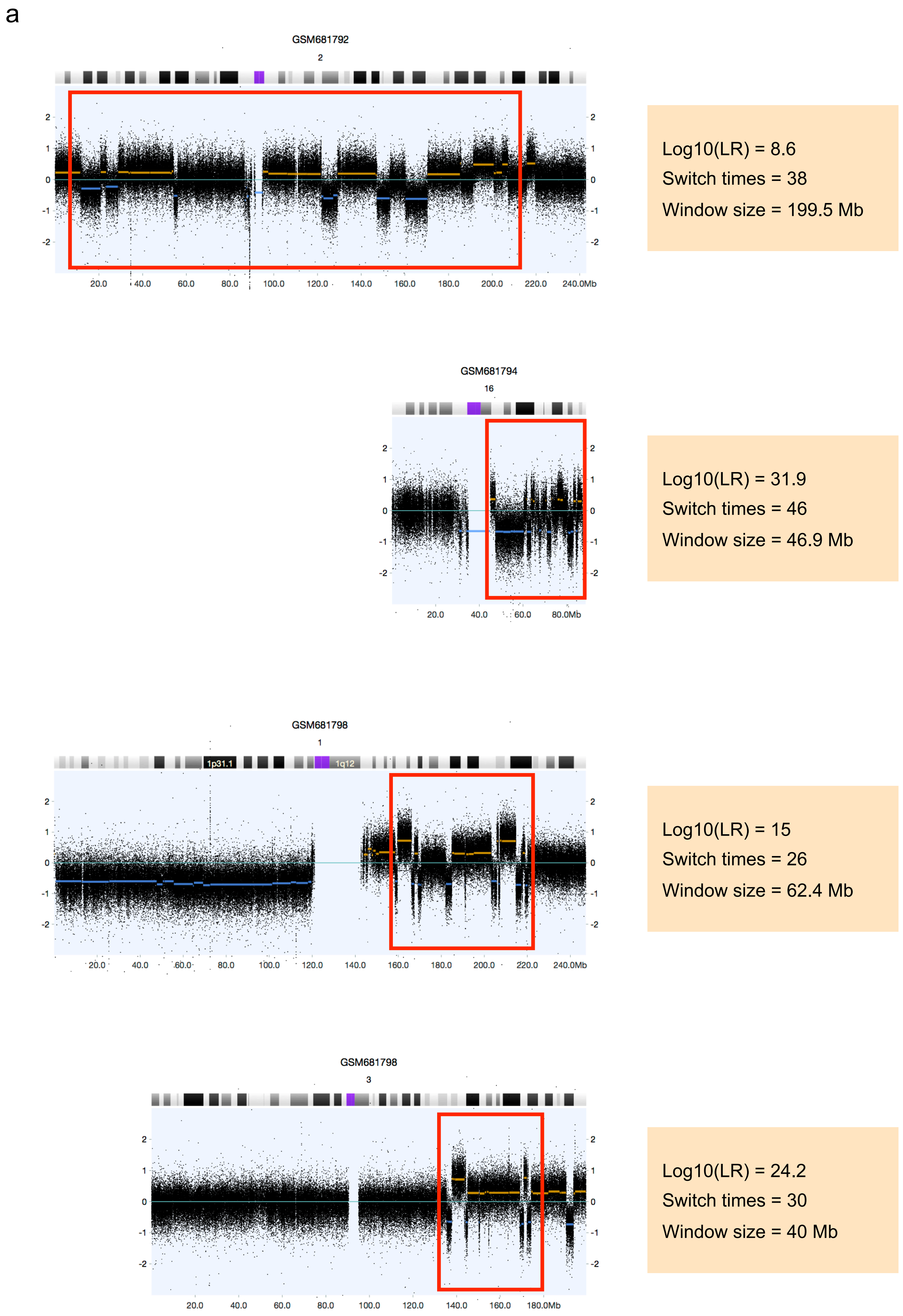
a
GSM681792
2
20.0 40.0 60.0 80.0 100.0 120.0 140.0 160.0 180.0 200.0 220.0 240.0Mb
Log10(LR) = 8.6
Switch times = 38
Window size = 199.5 Mb
GSM681794
16
20.0 40.0 60.0 80.0Mb
Log10(LR) = 31.9
Switch times = 46
Window size = 46.9 Mb
GSM681798
1
1p31.1
1q12
20.0 40.0 60.0 80.0 100.0 120.0 140.0 160.0 180.0 200.0 220.0 240.0Mb
Log10(LR) = 15
Switch times = 26
Window size = 62.4 Mb
GSM681798
3
20.0 40.0 60.0 80.0 100.0 120.0 140.0 160.0 180.0Mb
Log10(LR) = 24.2
Switch times = 30
Window size = 40 Mb

b

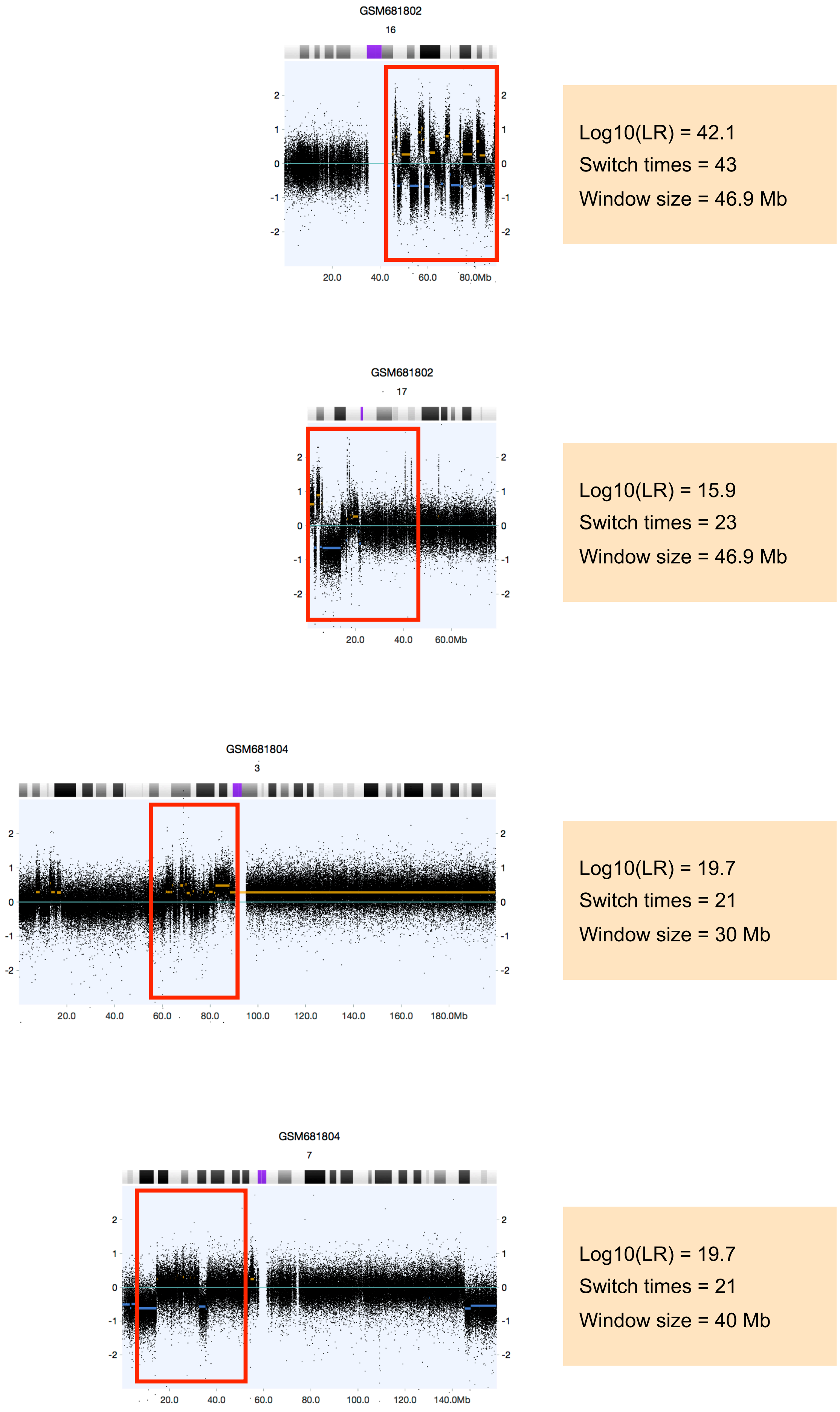
GSM681802
16
20.0
40.0
60.0
80.0Mb
Log10(LR) = 42.1
Switch times = 43
Window size = 46.9 Mb
GSM681802
17
20.0
40.0
60.0Mb
Log10(LR) = 15.9
Switch times = 23
Window size = 46.9 Mb
GSM681804
3
20.0
40.0
60.0
80.0
100.0
120.0
140.0
160.0
180.0Mb
Log10(LR) = 19.7
Switch times = 21
Window size = 30 Mb
GSM681804
7
20.0
40.0
60.0
80.0
100.0
120.0
140.0Mb
Log10(LR) = 19.7
Switch times = 21
Window size = 40 Mb

c

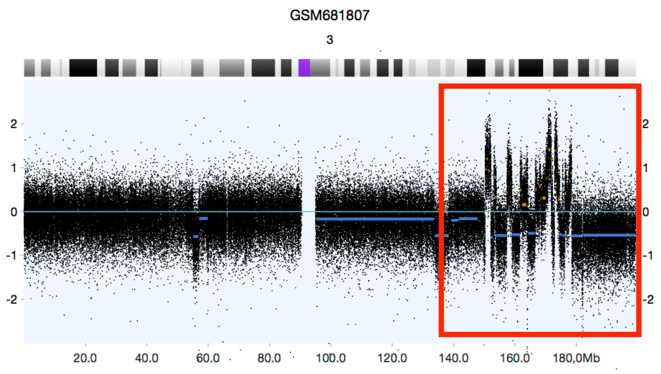

Log10(LR) = 58.4
Switch times = 61
Window size = 49.7 Mb

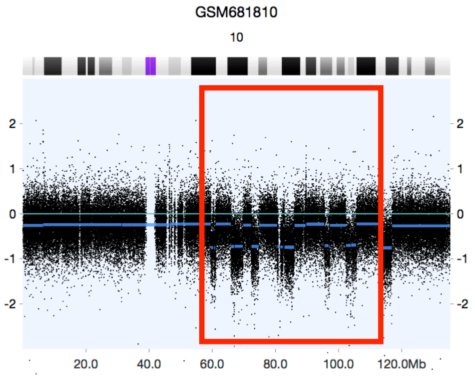

Log10(LR) = 13.7
Switch times = 21
Window size = 46.9 Mb

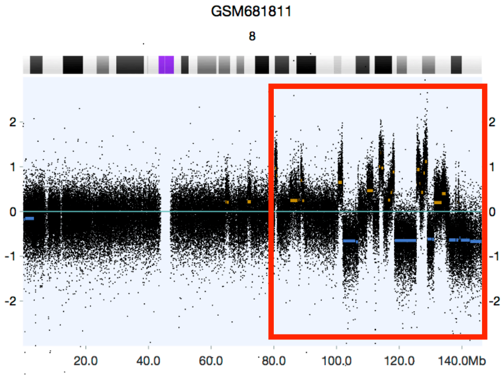

Log10(LR) = 57.7
Switch times = 60
Window size = 62.4 Mb

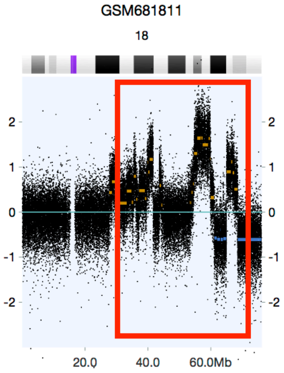

Log10(LR) = 26.7
Switch times = 26
Window size = 30 Mb

d

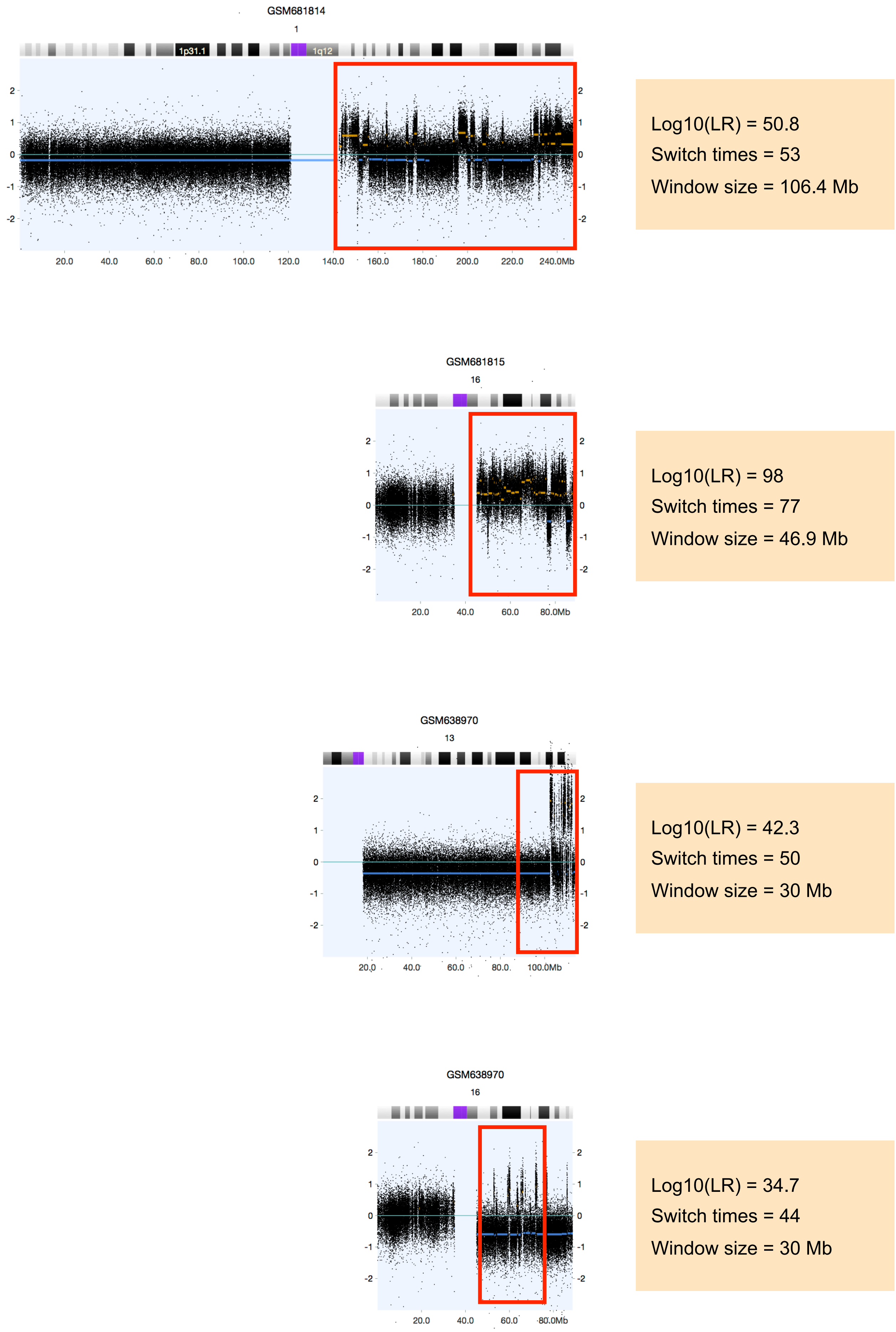

GSM681814
1
1p31.1
1q12
Log10(LR) = 50.8
Switch times = 53
Window size = 106.4 Mb
GSM681815
16
Log10(LR) = 98
Switch times = 77
Window size = 46.9 Mb
GSM638970
13
Log10(LR) = 42.3
Switch times = 50
Window size = 30 Mb
GSM638970
16
Log10(LR) = 34.7
Switch times = 44
Window size = 30 Mb

e

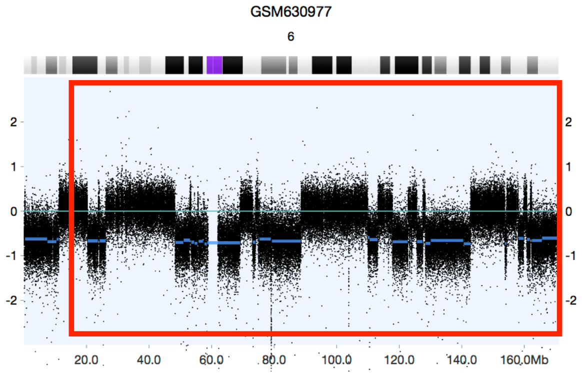

Log10(LR) = 31.8
Switch times = 51
Window size = 146.3 Mb

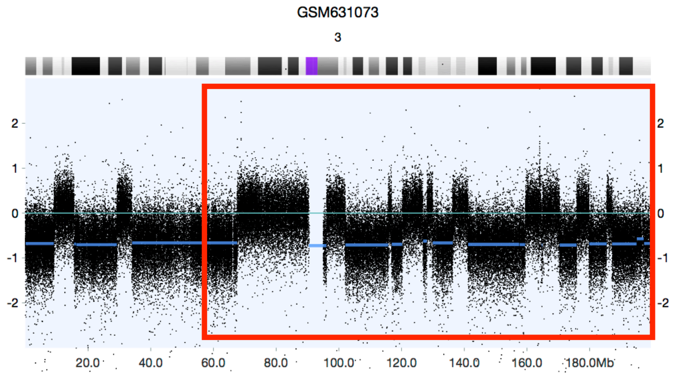

Log10(LR) = 8.8
Switch times = 24
Window size = 132.3 Mb

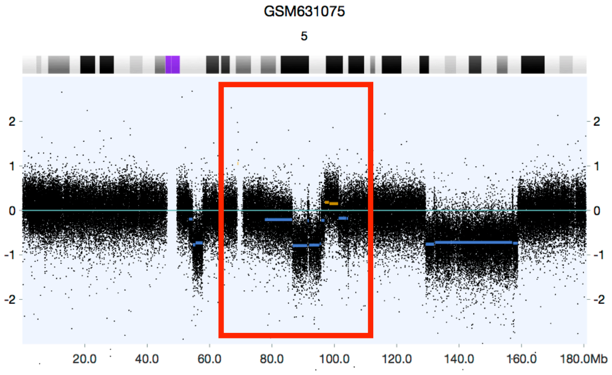

Log10(LR) = 10.7
Switch times = 14
Window size = 40 Mb

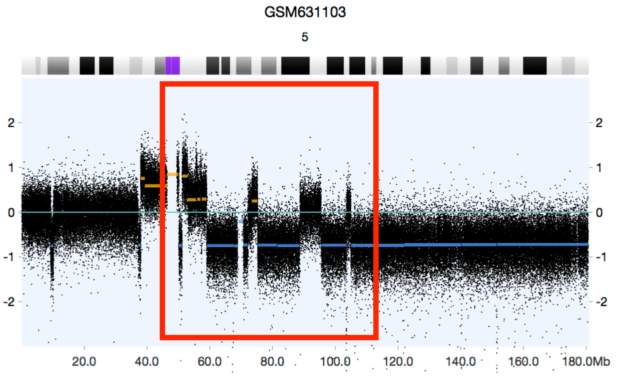

Log10(LR) = 18.4
Switch times = 25
Window size = 62.4 Mb

f

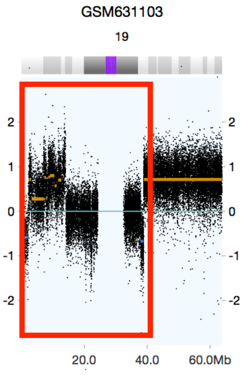

Log10(LR) = 28.7
Switch times = 27
Window size = 40 Mb

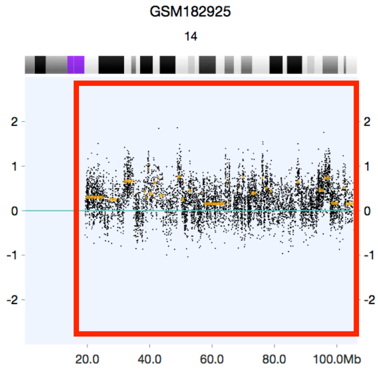

Log10(LR) = 57.7
Switch times = 51
Window size = 88.8 Mb

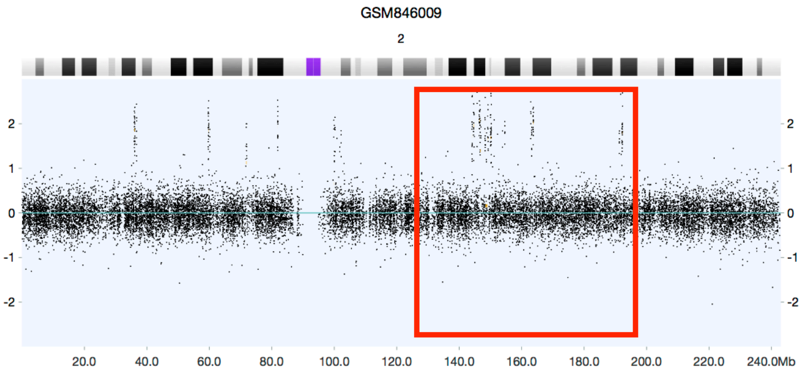

Log10(LR) = 34
Switch times = 30
Window size = 62.4 Mb

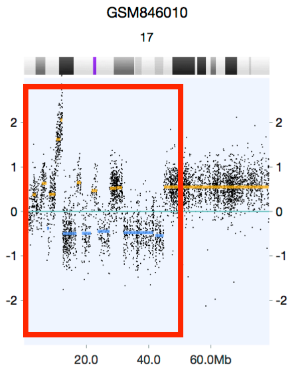

Log10(LR) = 16.4
Switch times = 18
Window size = 49.7 Mb

g

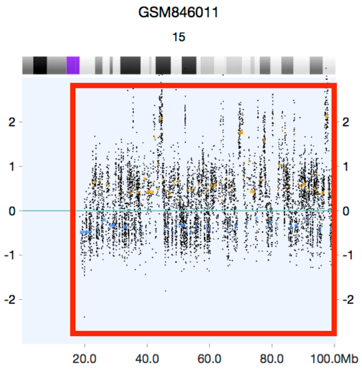

Log10(LR) = 240
Switch times = 186
Window size = 78.8 Mb

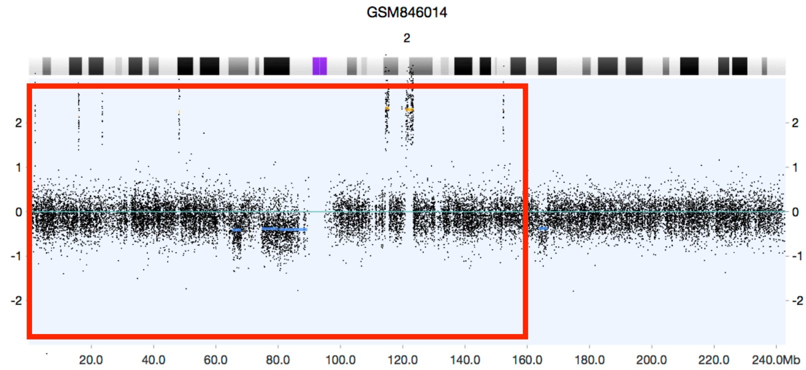

Log10(LR) = 11.1
Switch times = 21
Window size = 158.8 Mb

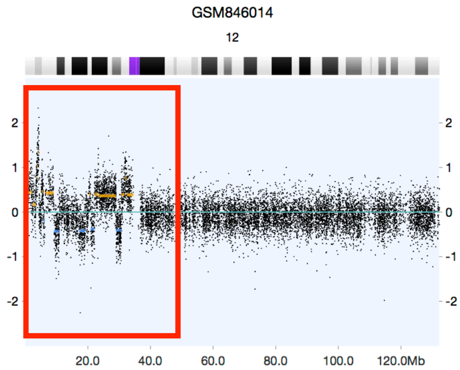

Log10(LR) = 25.1
Switch times = 24
Window size = 46.9 Mb

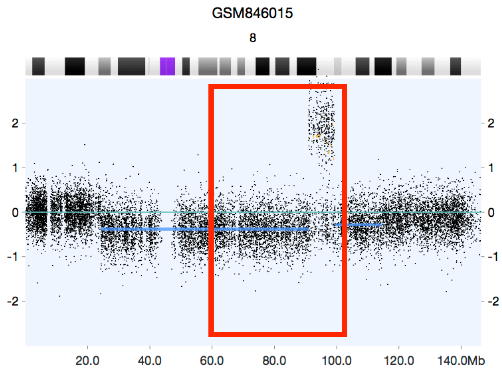

Log10(LR) = 48.5
Switch times = 38
Window size = 40 Mb

h

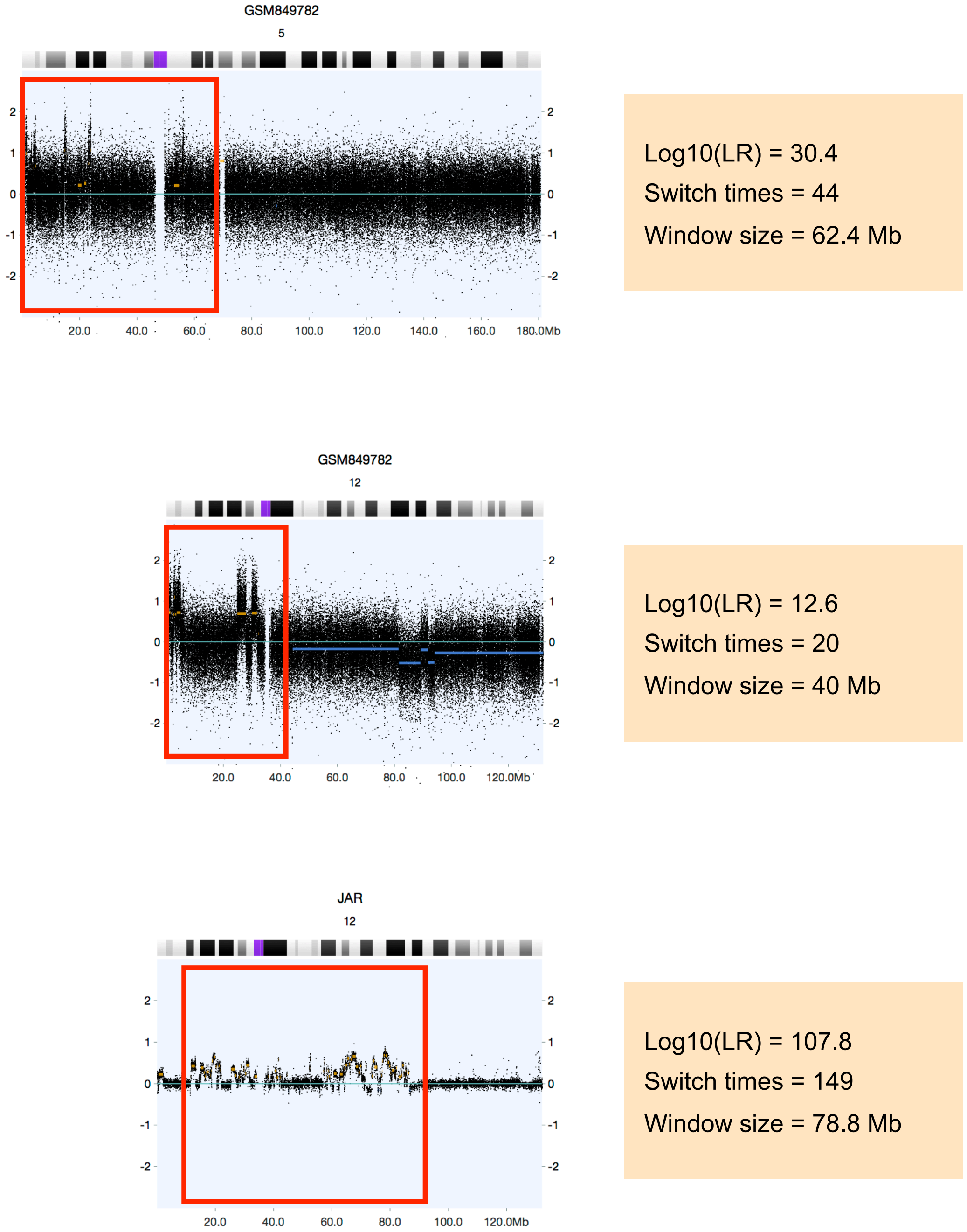

Figure S2. The positive training set and CTLP detection algorithm performances. The red rectangles are chromothripsis-like regions identified by scan-statistic. For each plot, the parameters and corresponding values are shown in orange boxes. The schema of the chromosome is the same as in Figure 2.

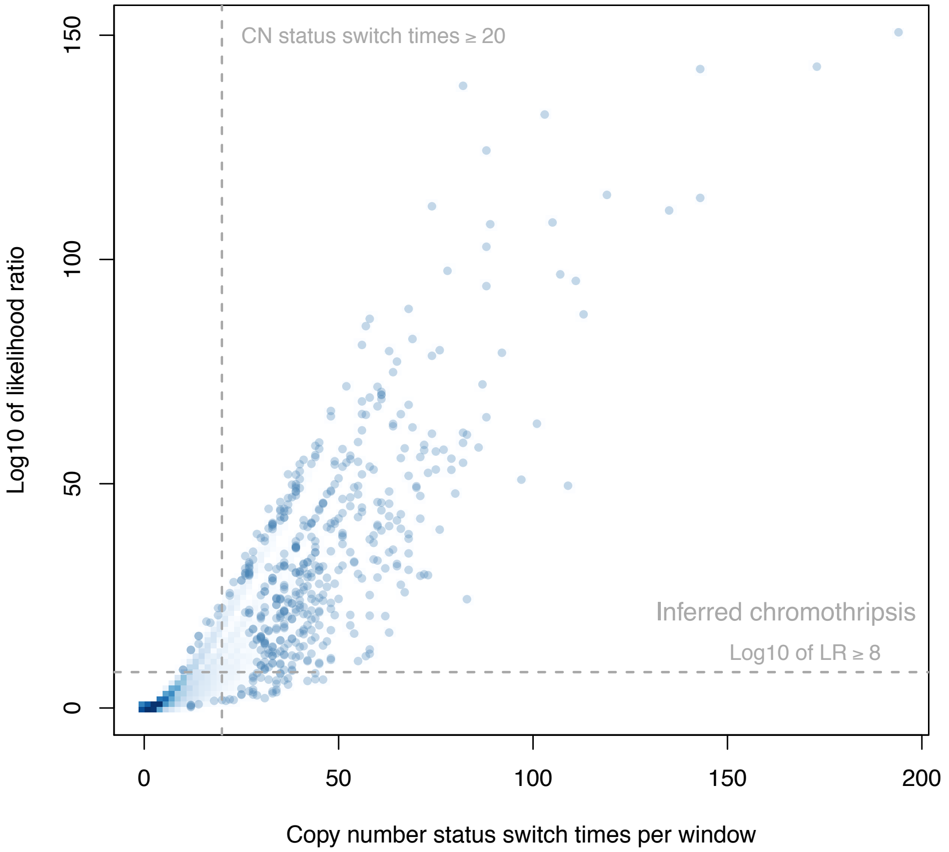

Figure S3. Scatter plot of CTLP candidates. For each chromosome of the input dataset, the window with the highest likelihood ratio was considered as a CTLP candidate. The selected thresholds are indicated with dashed lines. The candidates falling in the upper right area are inferred CTLP cases.

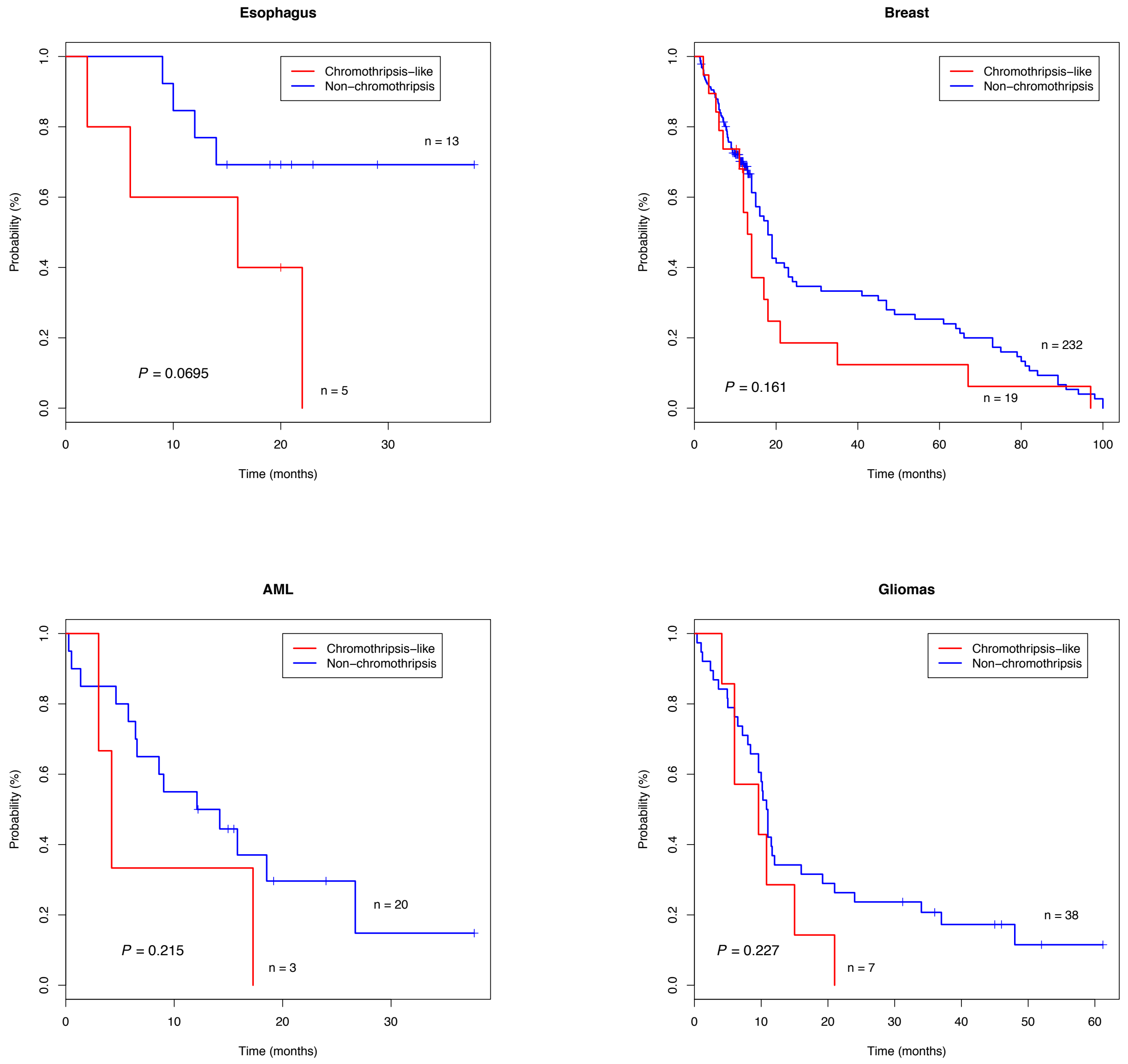

Figure S4. Kaplan-Meier survival curves for CTLP versus non-CTLP cases in specific cancer types.

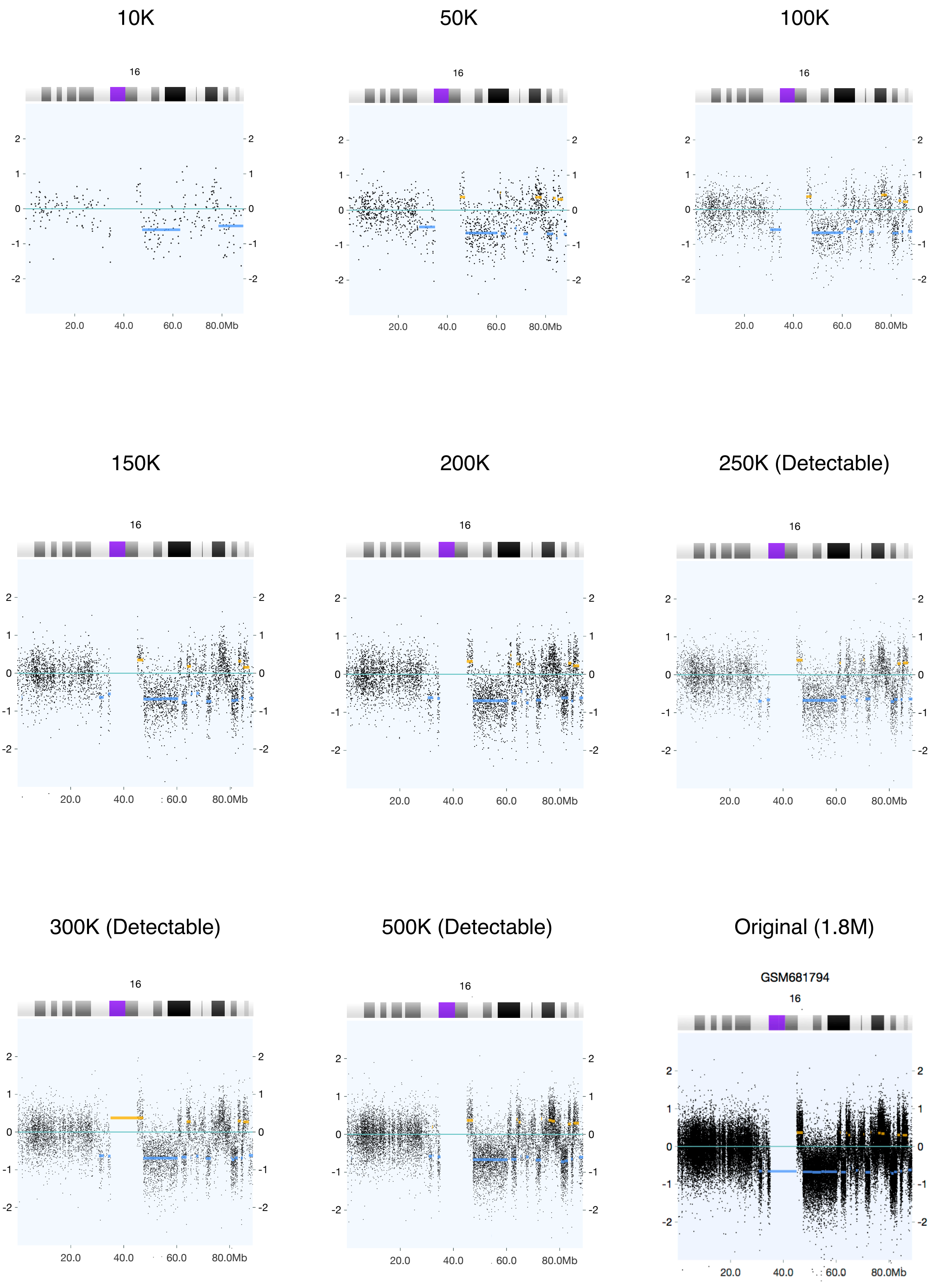

Figure S5. An example of the platform resolution based simulation from Affymetrix SNP6 array (1.8M). Chromosome 16 of GSM681794 is a reported chromothripsis event in multiple myeloma. The number above each plot represents the number of probes randomly selected from the original probe set. In this sample, the CTLP pattern is detectable starting at 250k probes.

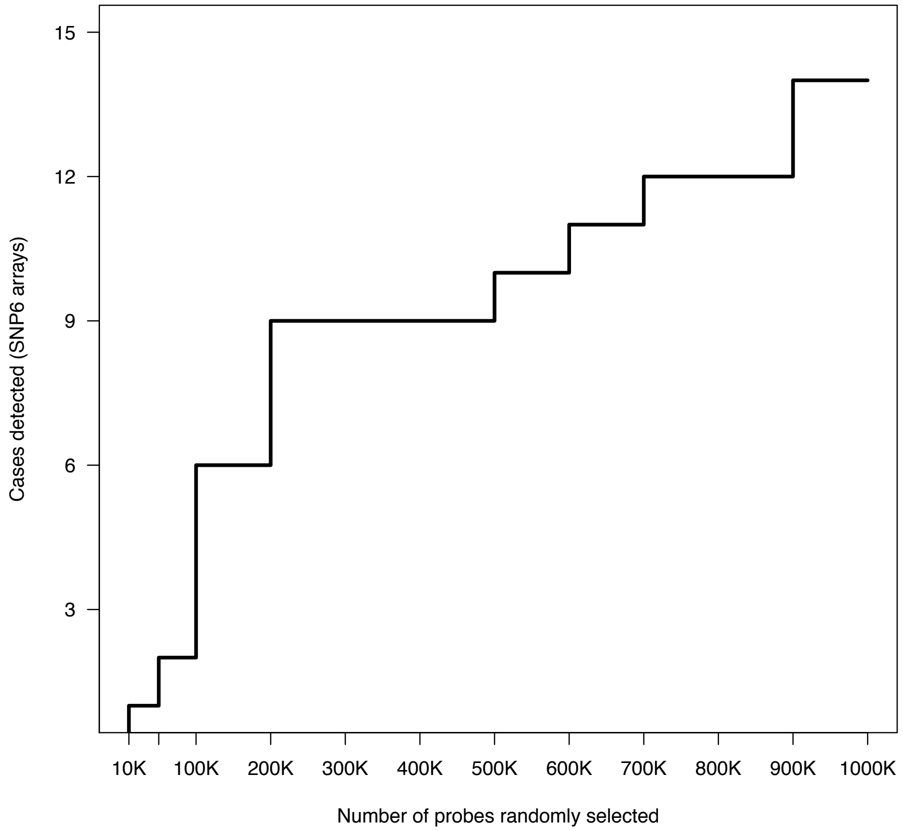

Figure S6. CTLP detection sensitivity of simulated platform resolutions. All the 15 chromothripsis chromosomes are from the positive training set analyzed using Affymetrix SNP6 platform.

Table S1. Overview of input dataset

Category	Array-level	Case-level
Total number	22347	18394
Series	402	397
Platform	190	185
Cancer type (ICD-O)	132	132
Cancer type (diagnostic group)	65	65
Source (primary)	19623	16309
Source (cell line)	2309	1714
Source (relapse)	75	75
Source (metastasis)	340	296

Table S7. Demographic and clinicopathologic characteristics of input and CTLP samples

Variable Name	Sample Number	Mean±SD/Median or %
**Input set**		
**Male**	1088	49.7%
**Age(year)**	2740	47.5 ± 25.7/55
**AJCC Stage**		
I	270	21.1%
II	331	25.9%
III	374	29.3%
IV	303	23.7%
**Grade**		
1	204	19.5%
2	435	41.7%
3	405	38.8%
**Tumor recurrence**	198	44.0%
**Follow up (month)**	1203	36.5 ± 34.6/26
**Event**		
Deceased	553	46.0%
Censored	650	54.0%
**CTLP**		
**Male**	46	32.6%
**Age(year)**	259	54.1 ± 20.7/59
**AJCC Stage**		
I	14	14.3%
II	36	36.7%
III	34	34.7%
IV	14	14.3%
**Grade**		
1	16	14.4%
2	49	44.1%
3	46	41.5%
**Tumor recurrence**	18	47.4%
**Follow up (month)**	72	32.9 ± 35.2/17.6
**Event**		
Deceased	46	63.9%
Censored	26	36.1%

† All information is based on the available clinical data

Table S8. Sizes of sliding windows for the scan-statistic based algorithm

Size ID	Size (Mb)	Corresponding chromosome
1	247.249	Chromosome 1
2	242.951	Chromosome 2
3	199.501	Chromosome 3
4	191.273	Chromosome 4
5	180.857	Chromosome 5
6	170.899	Chromosome 6
7	158.821	Chromosome 7
8	146.274	Chromosome 8
9	140.273	Chromosome 9
10	135.374	Chromosome 10
11	134.452	Chromosome 11
12	132.349	Chromosome 12
13	114.142	Chromosome 13
14	106.368	Chromosome 14
15	100.338	Chromosome 15
16	88.827	Chromosome 16
17	78.774	Chromosome 17
18	76.117	Chromosome 18
19	63.811	Chromosome 19
20	62.435	Chromosome 20
21	46.944	Chromosome 21
22	49.691	Chromosome 22
23	40	NA
24	30	NA
